# A novel theory of rapid eye movement sleep as an integral component of the seasonal timekeeping apparatus

**DOI:** 10.3389/fnins.2026.1838966

**Published:** 2026-05-26

**Authors:** Martin N. Raitiere

**Affiliations:** Department of Psychiatry (Retired), Providence St. Vincent Medical Center, Portland, OR, United States

**Keywords:** bed nucleus of stria terminalis, central extended amygdala, luxotonic signal, neurotensin, photoperiodism, REM sleep, sleep homeostasis

## Abstract

Although we have an increasingly sure grasp of much of its proximate circuitry, we continue to lack the ultimate physiological rationale of rapid eye movement (REM) sleep. In this paper, we propose that REM sleep derives both its proximate mechanisms as well as its ultimate cause from photoperiodism. (This refers to the means whereby many organisms translate information about day length into appropriately timed physiological adjustments ensuring their survival through the most challenging season—usually winter). First, the REM sleep interval serves, we suggest, as a sampling device attuned to a particular species of sidereal signal that materializes only in the crepuscular intervals of the day (when light slowly changes place with darkness) and that becomes fully available to the animal only in the shorter days (SDs) of the year. Second, REM sleep serves as an interval timer sensitive to the duration between shorter vs. longer phasic REM episodes, a distinction which a defined set of astrocytes then translates into that between, respectively, a temporal interval incapable of supporting aerobic glycolysis (AG) vs. one fully capable of doing so. Accordingly lactate, the product of AG, functions as a SD-specific signal triggering a behavioral, metabolic, and neuroprotective/neurogenetic program allowing the animal to survive winter. Outlined is the CNS seasonal module responsible for recognizing the lactate signal and disseminating it through the seasonal animal. This includes a novel photoperiodic role for the central extended amygdala and in particular the bed nucleus of the stria terminalis (BNST). Our model clarifies many different aspects of the REM sleep/seasonal amalgam including its coopting of basic arousal circuitry so as to support behavioral bistability, a key feature of the photoperiodic organism. Thus a remarkable but heretofore poorly understood phenomenon, a phase of hyperarousal preceding the descent into involution, falls into place as part of the strategy for surviving winter. Finally, our hypothesis is concordant with recent evidence demonstrating that the gene set subserving so-called lactate-mediated neural plasticity emerged well before that supporting traditional (explicit) memory, a specialty of mammals and their hippocampal tissue.

## 1 Introduction

Ever since its discovery (Aserinsky and Kleitman, 1953), the stage of rapid-eye-movement (REM) sleep, so named after the eye movements (REMs proper) that comprise one of its key phasic features, has posed something of a puzzle for modern neurobiology. Although REM sleep has been amply attested in nearly all mammals and birds (not to mention convincing prodromal forms in certain reptiles, amphibians, and invertebrates) (Blumberg et al., 2020; Jaggard et al., 2021; Rattenborg and Ungurean, 2023), its ultimate physiological rationale remains stubbornly unclear. We have, to be sure, benefited immensely from work in the past two decades on the neural circuitry subserving REM sleep. But even the remarkably granular discoveries regarding the pathways, neurotransmitters, and neuromodulators supporting REM sleep have not yet eventuated in a clear picture of its fundamental rationale. Another way of putting this is to suggest that the study of REM sleep has yet convincingly to cross over from proximate into ultimate levels of causation (Tinbergen, 1963; Nesse, 2019). We possess many answers as to where, when, and how, but few as to why.

In this paper I propose a hypothesis that offers a largely novel ultimate rationale for REM sleep. The approach taken here involves cross-fertilization between two research domains, namely those of REM sleep and photoperiodism, which with few exceptions have operated independently of each other. Photoperiodism refers to the phenomenon of an organism’s modifying its biology and behavior in response to the seasonally changing ratio between the illuminated (photophase) versus the dark (scotophase) portions of the day-night cycle. Having perceived such a change, the animal then instigates a species-specific program maximizing its chances for surviving the harshest season (generally winter). Thus classic smaller photoperiodic animals such as the Syrian hamster respond to the shorter days (SDs) of autumn and winter by developing, over 8–12 weeks, a striking involutional state. Such a state, when fully realized, eventuates in near-total abolition of sexual and reproductive function as well as torpor or hibernation, a response developing in perfect temporal synchrony with SDs and permitting the organism to survive the winter. This extraordinary alignment of physiology to the seasons is generally felt to represent an ultimate organizing rationale or cause behind the many proximate factors that can be identified within photoperiodism (Stevenson et al., 2022). In this paper we advance the hypothesis that REM sleep not only illuminates multiple proximate components of, but also borrows its ultimate evolutionary rationale and origin from, photoperiodism.

For reasons pertaining in part to the circumstances of its discovery in 1953, REM sleep for much of the second half of the 20th and into the current century was frequently associated with dreaming (e.g., Siclari et al., 2017) and, primarily through a link with the consolidation theory of learned experience, with the hippocampus (e.g., Hutchison and Rathore, 2015). However a very different approach to the delineation of REM sleep substrates had gotten underway in the 1960s with Michel Jouvet and his students in Lyon (Jones, 2018). By the end of the century his group had amassed, and in the current century its descendants continue to gather, much evidence implicating the brainstem, and specifically the mesopontine tegmentum, in the generation of REM sleep (e.g., Sapin et al., 2009). These results, along with those of other groups including Clifford Saper’s in Cambridge, Mass. (e.g., Saper et al., 2010), have lent credence to the notion that REM sleep and dreaming are dissociable states, the former originating with brainstem activity and the latter centering in forebrain, particularly hippocampal, tissue (Solms, 2000). Yet while yielding an impressive roadmap of brainstem centers controlling the generation of REM sleep, the Lyon and Cambridge groups as well as others with a similar focus have yet to unveil a genuinely ultimate cause for that phase of sleep. However if we inspect this roadmap through a lens provided by seasonal work, we find that it is rife with clues suggesting a role for the mesopontine tegmentum and loci rostral and caudal to it in the detection of changes in photoperiod—the critical evidential base which the animal requires in order to adjust its physiology to the seasons.

Our hypothesis does not require abandoning the view that REM sleep and the hippocampus play a role in the consolidation of memories of a particular kind, those involving explicit or consciously mediated experiences. [In non-human vertebrates with a hippocampus, this capacity subsumes spatial learning and memory (Othman et al., 2022)]. However we shall propose that explicit memory represents an evolutionary derivative of an earlier memorial function, implicit in nature and affiliated with seasonal physiology. This primordial function, termed photoperiodic memory (Prendergast et al., 2000), refers to the seasonal animal’s capacity for integrating a record not merely of one but of a series of recently experienced day lengths. Such an integrative (and in effect memorial) feature comprises an absolute requirement of photoperiodism if only because, each day length occurring twice in the calendar year, the organism must be able to distinguish whether a given day length is embedded within a progressively shortening series (in which case the animal will initiate its involutional response) vs. one of progressively longer days (LDs) (in which case it will continue to maintain its sexual and reproductive function). We shall thus present a case for the specialized memorial relevance of the seasonal substrate. What is known of that substrate currently? With qualifications to be noted below, this has traditionally been viewed as largely coincident with tissues responsible for the ‘readout’ of the melatonin signal, a well-established hormonal proxy for day length. These include primarily hypothalamic nuclei, i.e., the dorsomedial nucleus (DMN; Maywood et al., 1996; Leitner and Bartness, 2011; Jarjisian et al., 2013, 2015), the ventromedial nucleus (VMN; Maywood and Hastings, 1995; Bae et al., 1999), and (indirectly perhaps) the lateral hypothalamic area (LHA; Leach et al., 2013; Deats et al., 2014). Yet certain aspects of mammalian photoperiodism remain melatonin-independent (Yasuo et al., 2008; Otsuka et al., 2012).

This puzzle as well as others to be mentioned below may be resolved once we extend the seasonal substrate to include loci beyond the hypothalamus but related to it *rostrally* within the basal forebrain (especially the anterior bed nucleus of the stria terminalis [BNST; Raitiere et al., 1997]) and *caudally* (i.e., within the brainstem below the hypothalamus). Such an extension in both directions yields a basal forebrain- brainstem-based system displaying strong interconnections amongst different levels of the neuraxis *excluding the hippocampus*. The claims advanced here are (a) that such a hippocampus-exclusive continuum, one that began to coalesce prior to the appearance of tetrapods (Moreno et al., 2012; Lemaire et al., 2021), was early on adapted to serve in a seasonal network; (b) that a primitive form of REM sleep developed concurrently within that evolving pre-tetrapod network; (c) that the astrocyte, a cell type cognizable in its modern functions in fishes (Chen et al., 2020) and reptiles (Falcone, 2022), assumed a central role in the seasonal network in part by hosting aerobic glycolysis (AG) and consequently generating lactate; (d) that whereas the seasonal module inclusive of proto-REM sleep was adopted by mammals, in the process gaining a degree of connectivity with the hippocampus, it may in mammals nonetheless be dissected *functionally* from hippocampal capacities such as explicit memory. We propose to show how such an extrahippocampal continuum apprehends and responds to photoperiodic change by way of its capacity for REM sleep.

The notion of an astrocyte- and lactate-mediated as well as extrahippocampal form of memory has recently gained genetic support. Astrocytes and their lactate product entered neuroscience discourse in a decisive manner in the first two decades of the 21st century. Beginning with the studies of Marie Gibbs (Hertz et al., 2013), a number of authors including Pierre Magistretti developed evidence that traditional (explicit) memory, a quintessentially although not exclusively mammalian capacity, requires mediation by the astrocyte-based AG cascade productive of lactate (Gibbs et al., 2006; Suzuki et al., 2011; Hertz et al., 2013; Steinman et al., 2016; Alberini et al., 2018). Thus, by way of research disproportionately based on birds and mammals, a form of CNS plasticity eventually termed lactate-mediated neural plasticity (LMNP) secured a position as subserving hippocampal memory specifically. However as recently as 2022 a study authored by Magistretti among others complicated matters: Bajaffer et al. found that the gene set supporting LMNP evolved prior to that supporting explicit memory. (The two sets, each of which in both mice and men number over 300 genes, share 13 genes) (Bajaffer et al., 2022). Whereas *both* sets made “a leaping increase…just before the emergence of vertebrates” (Bajaffer et al., 2022, p. 5/9), the LMNP constellation as distinct from the memory set could be detected in many earlier organisms including classic chordates such as *Ciona* and *Amphioxus*. Interestingly Bajaffer et al. did not identify the LMNP set with a *global* environmental challenge to which it comprised the response; they nonetheless cautiously inferred the likelihood of “an unidentified system that links the memory and the LMNP systems” (Bajaffer et al., 2022, p. 2/9). We propose here that photoperiodism, largely realized in the earlier organisms and still quite discernable in the later ones including mammals, supplies the linking system. Thus as a through-line marking the proposed photoperiodic/proto-REM or REM sleep package whether in the earlier or the later animals, we shall offer AG, its lactate product, and LMNP.

Some remarks on the limits of our presentation may be helpful. Given our interest in the relationship between the LMNP vs. the traditional memory systems, we try to strike a balance between life forms illustrating each of these, i.e., the (largely) pre-mammalian vs. the (largely) mammalian domains, respectively. Examples from mammals will include both classic photoperiodic species including hamsters and that fraction of human beings which exhibits seasonal affective disorder (SAD), generally considered a residuum of animal photoperiodism (Rosenthal et al., 1984). (Since SAD is strongly affiliated with bipolar disorder [BD], especially but not only BD type 2 [Akhter et al., 2013; Geoffroy et al., 2014; Maruani et al., 2018], we term the relevant human syndrome SAD/BD). As for pre-mammalian organisms, we shall draw upon reptiles, fishes, and non-vertebrate chordates. Within both of these large categories, we shall be concerned with the response of photoperiodic organisms to the SDs of autumn and winter. (This excludes the intriguing problem of so-called spontaneous recrudescence in spring). We do not routinely say much about non-REM sleep; nevertheless it should be understood throughout to provide significant support to REM sleep. The thermoregulatory axis, admittedly an integral part of the seasonal animal’s capacity for dealing with winter, does not enter until close to the end. Serotonin, ATP, and adenosine, also integral to photoperiodism, go unmentioned. Other intentional omissions will be mentioned where appropriate.

## 2 The proposed photoperiodic/REM sleep substrate

**Figure 1:**
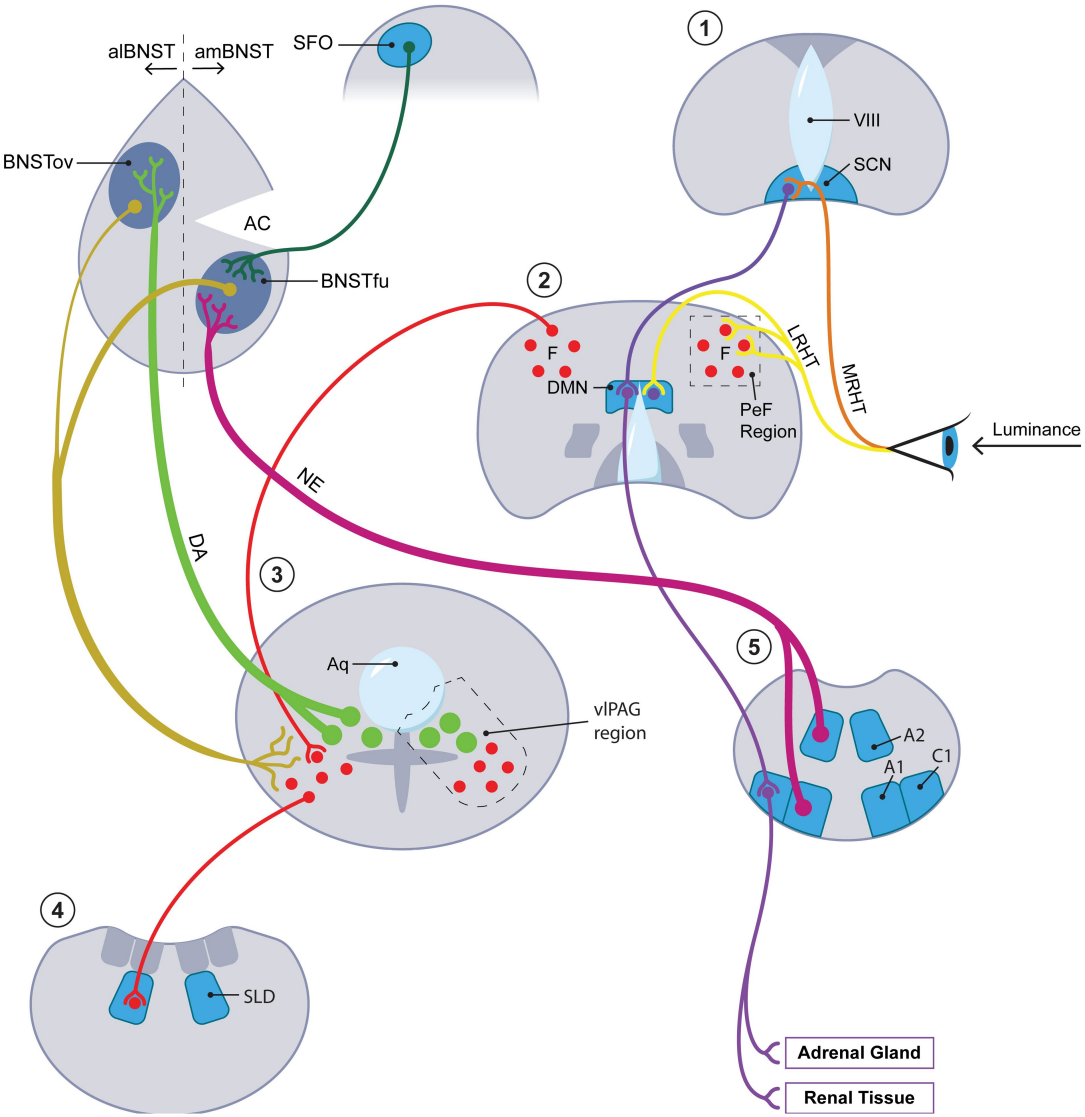
The hypothesized photoperiodic/REM sleep network. The diagonal axis going from lower left to upper right features four coronal sections with the most rostral stationed at the upper right. A fifth, most caudal in the series, is placed at the lower right of the figure. Each of the five coronal sections is indicated by a circled numeral (from rostral to caudal): 1 = hypothalamus at level of SCN (for abbreviations, see below); 2 = hypothalamus at level of DMN; 3 = periaqueductal gray region at level of DA A10dc neurons; 4 = pons at level of SLD; 5 = medulla at A1/C1 level. At level 3, closed green circles represent DA A10dc neurons; closed red circles, GABA neurons. At upper left, the diagram concentrates upon the anterior BNST but also includes the SFO as representative of all of the CVOs (contribution of CVOs to photoperiodism to be addressed later). Terminology of BNST subnuclei generally derives from Swanson and colleagues (e.g., Dong et al., 2001) with one exception: the division here of the anterior BNST into anterolateral vs. anteromedial sectors is not identical with theirs. Figure proper reproduced from Raitiere, 2022, with revised caption. All supporting references are available in text (primarily section 2; for the efferent from the DMN to the A1 cell cluster, one which is likely subject to change under seasonal pressure, see Figure 3 and caption). Two loci not included here, the lateral habenula and the rostromedial mesopontine tegmental nucleus, will be added with full discussion later. Note the exclusion from this figure of the PFC, a region that enters the seasonal module with mammals: as per claim (d) in section 1 of our text, the PFC integrates that module for its own purposes without overriding its seasonal capacities. Limitations of space preclude a full discussion of that process of integration. IIIV, 3rd ventricle; A1, A1 noradrenergic cell group; A2, A2 noradrenergic cell group; AC, anterior commissure; alBNST, anterolateral division of BNST; amBNST, anteromedial division of BNST; Aq, cerebral aqueduct; BNST, bed nucleus of stria terminalis; BNSTfu, fusiform subnucleus of BNST; BNSTov, oval subnucleus of BNST; C1, C1 adrenergic cell cluster; CVOs, circumventricular organs; DA, dopamine; DMN, dorsomedial nucleus of hypothalamus; LRHT, lateral RHT; MRHT, medial RHT; NE, norepinephrine; PeF, perifornical region of lateral hypothalamus; PFC, prefrontal cortex; RHT, retinohypothalamic tract; SCN, suprachiasmatic nucleus of hypothalamus; SFO, subfornical organ; SLD, sublaterodorsal nucleus; vlPAG, ventrolateral periaqueductal gray.

At the turn of the 21st century, the pioneering brainstem-based work originated by Michel Jouvet held sway within the field of REM sleep physiology. This highlighted in particular two partly cholinergic nuclei at the mesopontine tegmentum (the pedunculopontine tegmental nucleus [PPTg] and the laterodorsal tegmental nucleus [LDTg]). However more recent developments have distinctly broadened its neural substrate. This updated REM sleep substrate (one to which Jouvet’s descendants and Saper’s team among others have contributed much through newer, e.g., optogenetic means) now embraces significant roles for the medulla (Sapin et al., 2009; Clément et al., 2014; Weber et al., 2015; Schott et al., 2023) and for the prefrontal cortex (PFC; Hong et al., 2023) while incorporating as well hypothalamic nuclei already well established within the seasonal network including the DMN (Luppi et al., 2013) and LHA (Vetrivelan et al., 2016). Therefore whatever their *explicit* emphasis, these recent discoveries by students of REM sleep *implicitly* place that function *and* a generous portion of the seasonal substrate within the same continuum. In so doing, they license our initial rendering of a *unitary* photoperiodic/REM sleep network in Figure 1.

Several features of the network proposed in Figure 1 call for comment at the outset. What especially interests us in the seasonal module is its changing in synchrony with photoperiod—yet this is the aspect most difficult to capture in such a schematic diagram. We shall mention several dynamic aspects briefly here as a promissory note with explanation to follow. First, luminance itself will prove to be a major motor of such reconfiguration. A second such motor, the cholinergic system, will be discussed briefly where appropriate. Third, astrocytes, omitted from Figure 1, will serve variously to orchestrate change within the seasonal module, especially by way of housing AG and thus making possible LMNP. Fourth and finally, we shall develop the position that the central extended amygdala (CEA) and especially the BNST, included here, provides a major substrate for photoperiodic change.

It will be noted that our view of the CEA, or at least of its BNST portion, differs significantly from one has held sway for years. Almost from its inception, the CEA (Alheid et al., 1995) has been described as a circuit that deals with stress and its attendant anxiety. This position was enunciated clearly by Michael Davis and colleagues beginning in the 1990s (Davis et al., 1997) and it continues to be invoked regularly by those contributing to the recent explosive interest in the BNST (e.g., Marcinkiewcz et al., 2016; Snyder and Silberman, 2021; Showemimo et al., 2026; Sun et al., 2026). Now a stress-centered context generally implies (a) that the organism’s response *follows* a given stressor; (b) that the stressor in question is *incipiently if not fully present* to the animal or human; (c) that the organism’s affective response involves some form of hyperarousal. *While we do not challenge the bearing of the stress/anxiety context on the amygdaloid portion of the CEA, i.e., the central nucleus of the amygdala (A_ce_), we question here the applicability of that context to the BNST portion*. In our view, of the aforesaid conditions, only (c) applies to the BNST, and that partially: its participating in the hyperarousal of SD, as we shall see, does not follow a consciously appreciated stressor but rather precedes the winter ‘stressor’ that will materialize later on. For the photoperiodic mammal displays distinct neuroendocrine evidence of getting its involutional process underway by the first or second week of August, i.e., relatively early in naturalistic SD and thus well before the onset of winter (Liu and Burbach, 1987; though a fascinating topic, the manner in which the summer rise in vasopressin and oxytocin prepares at some distance for the involutional phase cannot be elucidated here). Now while the Davis group saw stress as relevant equally to the BNST and A_ce_, they presciently characterized the BNST as working within a significantly longer temporal framework than the A_ce_ (Davis et al., 1997; Walker et al., 2003, 2009). On that score we agree emphatically, differing only in proposing that the longer framework is specifically the seasonal one.

A recently published fMRI study of humans processing a threat anticipation paradigm finds that, within strictly defined limits, the BNST and A_ce_ display suggestions of “regional equivalence,” thereby undercutting, the authors claim, the Davis paradigm (Cornwell et al., 2025). However the authors compare responses to two highly interrelated species of threat insofar as both are defined as fully conscious and subject to capture by the same fMRI interrogation. Their topic and methodology does not purport to investigate the months-long *and largely subliminal* processing of seasonal developments which is central to our hypothesis. A clear functional distinction between the BNST and the A_ce_ would emerge, we suspect, with study of these CEA components under differing photoperiods. Absent such an approach, the findings of Cornwell et al. must remain neutral with respect to our hypothesis.

Although organic change over the solar year involves genomic plasticity, i.e., modification over SD of the expressed genome (e.g., Dulcis et al., 2013; Petri et al., 2016; Porcu et al., 2022), a more appropriate genre of CNS change for our purposes is that subsumed under the term phenotypic plasticity. This concerns strategies centering on what Linda Weiss has aptly termed sensory ecology (Weiss, 2018; Weiss et al., 2022). As for the sensory ecology of REM sleep itself, this will provide the core of our case for its intimate link with seasonal physiology: we shall argue that REM sleep serves as a recurrent probe for the very environmental signal, a sidereal one, that is essential to photoperiodism. In addition, REM sleep will be shown to introduce a number of the engines by which that sidereal change is translated into organismal. Such translation capacity rests at least in part on REM sleep’s association with both acetylcholine (ACh) release and nitric oxide (NO) generation (Leonard and Lydic, 1997), the latter in turn contextually stimulating the AG cascade and consequently lactate production in astrocytes (Brix et al., 2012; San Martín et al., 2017). (The context inviting such a cascade will be defined below). Working alongside each other, these molecular mediators, we shall suggest, promote both the proximate mechanisms and the ultimate cause, namely species survival in a seasonally changing climate, of photoperiodism.

Before further developing the seasonal/REM sleep amalgam, we address two additional topics that warrant discussion insofar as either frequently misunderstood or largely new to photoperiodism.

## 3 Problematic aspects

### 3.1 Why anchor the narrative on the autumn equinox?

We submit that until fairly recently seasonal plasticity on the behavioral level has been frequently misunderstood or collapsed into a uniform series of events. For the LD → SD transition has often been referenced as a slow and linear process of involution down to its terminus in torpor or hibernation. However the phrase ‘SD involution,’ although useful as shorthand, elides a key part of the seasonal narrative. In the 21st century, evidence has accrued that the early weeks of SD may include features of irritability and outright aggressiveness—indices of behavioral *hyperarousal*, the exact opposite of the hypoarousal defining the conclusion of the process. To be fully patent, such hyperarousal may call for some degree of unmasking through modification of hormonal and other variables (e.g., Jasnow et al., 2000, 2002; Demas et al., 2004; Scotti et al., 2008; Munley et al., 2022). No such unmasking, however, is necessary in the case of the intense and (for once) fully conscious hyperarousal featured by seasonal organisms in the vicinity of the autumn equinox. Such flagrant hyperarousal is a key attribute, in larger, SD-breeding quadrupeds, of the rut (Lincoln and Short, 1980); in migratory avian species, of the pre- and intra-migratory phase, the former termed *Zugunruhe* (e.g., Lupi et al., 2019); in human beings with SAD/BD, of the manic or hypomanic episodes which, as well documented, peak in the autumn (e.g., Maruani et al., 2018). In practically all of these cases, evolution has maintained a rough conjunction of hyperarousal with the autumn equinox. In the Soay sheep, for example, a primitive and unimproved breed now restricted to an island off the coast of Scotland, the rut, lasting through October and November, begins in late September (Lincoln and Short, 1980). The equinoctial hyperarousal may highlight specific features such as irritability and aggressiveness in the quadrupeds as well as in those affected humans displaying so-called dysphoric mania (McElroy and Keck, 2017); profound insomnia in migrating avian species as well as in manic-phase humans (Rattenborg et al., 2004); and hypersexuality in the quadrupeds intra-rut and frequently in the manic-phase human beings. One *proximate* reason for such centering of arousal on that equinoctial date is not far to seek: the *rate of change* of photoperiod is greatest at that juncture (Rosenthal et al., 2021). It is also important to note that hyperarousal as such is the primal feature; it may be dissociated from a particular use of that state such as migration; thus *Zugunruhe* obtains even in a nonmigratory bird (Helm and Gwinner, 2006).

Behavioral plasticity over the course of SD therefore involves excursions of arousal both above (most obviously at the autumn equinox) and below baseline (in the much longer post-equinoctial descent). Whatever later forays we make into seasonal plasticity at a molecular level must account for that complex (biphasic) behavioral picture. We belabor this point because it bears on the vexed question of how *daily* plasticity (the temporal dimension in which we find REM sleep) interdigitates with the seasonal form. One major step in resolving that issue, we suspect, would involve transcending the view that a single CNS locus could account for the entire arc of photoperiodism. That view, not surprisingly, rapidly gained traction in the wake of the 1972 discovery of the suprachiasmatic nucleus of the hypothalamus (SCN) as an apparently endogenous oscillator and presumptive “master clock” of mammalian circadian rhythms (Hendrickson et al., 1972; Moore and Lenn, 1972). From that complex story we are concerned only with the question whether such a perspective helped or hindered our understanding of photoperiodism. After all, a fully endogenous master oscillator would by definition be perfectly buffered against *any* extrinsic influence—including the change of seasons.

### 3.2 Seasonally relevant tissues beyond the suprachiasmatic nucleus

A number of different findings in the late 20th and early 21st centuries chipped away at, without quite dislodging, the conception of the SCN as master oscillator in nearly all domains including the seasonal. While some early studies within this time-period found that animals bearing complete lesions of the SCN obviated their species’ usual capacity to respond to a SD environment (Bartness et al., 1991; Hakim et al., 1991), other, more recent, work has highlighted a ‘federated’ organization wherein light (i.e., as luminance) can control peripheral clocks independently of the SCN (Husse et al., 2015; Astiz et al., 2019; Tsuno and Mieda, 2024). Furthermore, that lesioning of specific *extra-SCN* sites within our proposed substrate (particularly the VMN [e.g., Maywood and Hastings, 1995], the DMN [e.g., Maywood et al., 1996], and the BNST [Raitiere et al., 1997]) clearly compromises the SD response strongly suggests that these loci have a claim to photoperiodic credentials at least equaling that of the SCN. Each of them, in other words, comprises a necessary (although not a sufficient) seasonal node at least until proven otherwise. Many other examples of the *partial* independence (variable in degree) from the SCN of loci affiliated with the photoperiodic/REM sleep substrate have steadily accrued up to the present (e.g., Masubuchi et al., 2000; Carr et al., 2003; Yan et al., 2006; Ruan et al., 2012; Bautista et al., 2025). In any event, nothing in our position demands the *absolute* independence of the seasonal substrate from the SCN. *All that is required is what we shall call the openness of the specified ‘federated’ network to luminance—a position, incidentally, that need not place circadian rhythms as such at risk*. One of the shrewdest students of the role of circadian rhythmicity as a crucial feature of photoperiodism evidently did not believe that such rhythmicity demanded a single ‘master’ oscillator. Indeed from 1936 to as late as 1973 *after* the discovery of the largely endogenous rhythmicity of the SCN, E. Bünning regularly distinguished two capacities of light: that involving entrainment of a central oscillator by a *Zeitgeber* in the form of a short blast of morning light vs. that pertinent to photoperiodism implying determination of length of day (Bünning, 1936, 1973). Admittedly the former has everything to do with the SCN; the latter, for reasons to be clarified below, need not include the SCN on a regular basis. Indeed in certain species the SCN becomes inoperative in hibernation (Revel et al., 2007). Yet circadian rhythms survive robustly during torpor (Körtner and Geiser, 2000) and even in a number of hibernators the circadian system “appears to remain functional” (Zervanos et al., 2009, p. 103). Little in these and related works prohibits the inference of a gradual ‘handoff’ of circadian rhythmicity as SDs progress from the SCN to the ‘federated’ organization, equally competent with the SCN to maintain such rhythmicity.

For our purposes, surely the most relevant findings pertaining to photoperiodism began with the discovery that certain nonvisual projections from the retina entirely skirt the SCN. Thus whereas the medial branch of the RHT (MRHT) terminates in the SCN, the lateral branch (LRHT) avoids it entirely, targeting instead different seasonally relevant sectors such as the LHA and medial hypothalamus including the DMN (Johnson et al., 1988; Leak and Moore, 1997; Canteras et al., 2011). For some years the clinical relevance of these extra-SCN retinal projections was not apparent. That began to change about 2017 when R. Burstein’s group published evidence linking these projections to the autonomic and (unpleasant) affective responses to light in migraineurs (e.g., Noseda et al., 2017). (Migraine is a condition highly comorbid with SAD/BD [Leo and Singh, 2016] and one that itself displays seasonal modulation [Bekkelund et al., 2017]). These findings were of particular value for bringing nonvisual retinal connectivity into the orbit of *pro*nociceptive physiology; as such, they represent a counter-example to reports that bright light treatment for mood disorders has an *anti*nociceptive effect (Hu et al., 2022). Thus luminance itself has access to *both directions* of a dichotomy, i.e., modulation both above and below an arousal as well as an affective baseline. Yet whether such a bistable capacity could somehow be scaled up to drive the months-long canonical photoperiodic sequence remains to be seen.

This concludes our section 3 on problematic features within seasonal physiology. It is time to develop the case for REM sleep as an integral part of the seasonal package.

## 4 REM sleep plays approach-and-avoid with a crepuscule

**Figure 2:**
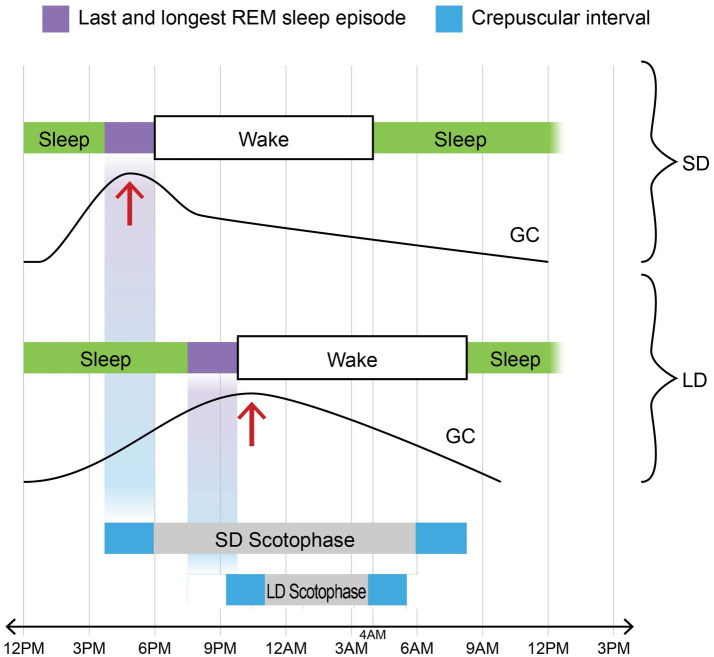
Consequences for REM sleep of engagement with the pre-waking crepuscule. This figure represents the case for a nocturnal animal; converting this into the case for the diurnal animal would require little more than changing the time axis. Two different kinds of chang in the relationship between the last REM sleep episode and the pre-waking crepuscule transpire as the organism in the wild transitions from a LD to a SD environment. **(a)** In LD the crepuscule barely intrudes on the last REM sleep interval; indeed if it does at all, it likely serves to cancel sleep, thus eliminating any overlap between twilight and REM sleep. As SDs progress, the crepuscular interval shifts slowly into the last REM episode, gradually losing (for unclear reasons) the capacity to awaken the animal; indeed it is replaced by the imperative to extend sleep. In the top third of the figure, we depict the consummation of this SD trend: the crepuscular interval is now roughly coterminous with the last REM sleep interval. If the animal should be capable of sensing luminance in that episode, then its favorite target under SD would seem to involve *luminance in transition* (see discussion in section 4). **(b)** The next relevant change marking the shift to SD involves the position of the glucocorticoid (GC) pulse acrophase, marked by a large red arrow, with respect to the last REM episode. Thus in LD the GC acrophase falls shortly after the animal has fully awakened. Medical textbooks routinely explain its post-waking location as addressing the stress, behavioral and metabolic, of the coming day: foodstuff must be found and ingested and the animal made ready to metabolize it. However in SD the GC pulse acrophase phase-advances with respect to the more inertial sleep–wake cycle, eventually falling inside the last segment of sleep, ending roughly in the middle of the last REM sleep episode. The functional implications of the GC pulse phase advance have generally been neglected. Exceptionally, two groups have made findings regarding this shift that bear on our overall hypothesis. Kanikowska et al. (2019), show that the SD seasonal rearrangement is not restricted to pathological variants of photoperiodism: it obtains in normal human beings (i.e., in a cohort assembled without exclusions for psychiatric illness). Pierre et al. (2018), add helpfully that the ensemble of hormonal oscillations in SD reveals more robust attachment of peripheral clocks to light signals than obtains in summer. Our own view of the ramifications of positioning the GC acrophase inside sleep in SD is given in section 4. Abbreviation not previously mentioned: GC, glucocorticoid; L: D, hours of photophase vs. hours of scotophase. Figure proper is reproduced with modifications from Raitiere (2022).

For both diurnal and nocturnal animals, a contingent relationship obtains over the seasons between their *organismal* times of early and late sleep on the one hand vs., on the other, a critical pair of *sidereal* variables, namely the dawn and dusk crepuscules in which light and darkness slowly change places. While each of the sidereal boundary zones occurs roughly at the beginning and the end of the organism’s sleep phase, the two elements comprising each of the resulting sidereal/organismal pairings do not have a fixed relationship to each other. Thus in Figure 2, we depict the manner in which the second crepuscule and the second of the two sleep–wake transitions play approach-and-avoid over the seasons.

How does the fluid relationship between the two components of our second couplet of durational intervals, one organismal (late sleep) and the other sidereal (the second crepuscule), bear on the animal’s response to SD? In LDs the late sleep interval intervenes marginally on the pre-waking crepuscule. Indeed if humans do awaken *as a result* of early light as suggested by many studies of the “cortisol awakening response” (e.g., Stalder et al., 2025), then this suggests that the LD norm cancels any possibility of sleep’s intruding more than minimally into the relevant crepuscule. Only as the days shorten through autumn do that late sleep segment (that is, the one containing the final REM sleep episode) and the pre-waking twilight begin decisively to approach each other and to overlap. Indeed at some date in SD the entire culminating REM sleep episode will have been shifted into the sidereal twilight interval, itself frequently coming to an end before the organism awakens. What does evolution gain from this gradual repositioning, in SD, of REM sleep within the second crepuscule?

The gain may come less from the phase-advance of REM sleep than from a concurrent augmentation and phase-advance of roughly equal magnitude, that of the glucocorticoid (GC) acrophase (see Figure 2). The latter is an underreported but likely common development in the SD mammal (Lemos et al., 2009), including, provocatively, a cohort of human beings without psychiatric illness in winter vs. summer (Kanikowska et al., 2019). The shift of the GC acrophase from early waking in the LDs of summer to late sleep in the SDs of autumn may result in the powerful augmentation by GCs themselves (Simard et al., 1999) of calcium fluxes within the seasonal module. A proposal for the connection between such calcium flux and augmented sensitivity to luminance will follow later on.

Yet the claim that the animal can engage *in any fashion* with an environmental variable while asleep may generate an objection. How could animals including human beings sense anything while in any phase of sleep? That mice and humans can discern modest degrees of luminance through closed eyelids (e.g., Figueiro, 2015) may not satisfy those who insist that perceptual processing requires full consciousness. (With his theory of unconscious inference, Helmholtz among others thought otherwise [Kihlstrom, 1987]). Partisans of dreaming as a quintessential aspect of REM sleep may help here: they have claimed that the individual in phasic REM sleep is directing his eyes toward an object that comprises an element in his dream sequence. We are skeptical with others of that assertion—how the dreamer could be attending in any traditional sense to a single discriminated object while his globes are phasically moving every 2–3 s or so defeats us. Yet we see no reason to doubt that dreaming figures *somehow* amongst REM sleep phenomena; indeed we welcome the efforts of its partisans to explore the possibility that the globes propelled by REMs are attending to *something*. Our position is that *whether or not* a dreamer is following an object dreamt, he is at the same time processing a datum truly obtaining in the sensory environment. It is the latter process, not the former, that demands our attention.

## 5 Late sleep and crepuscules: internal organization of each

Much of the discussion in the previous section was prefigured in the so-called external coincidence model originally proposed in Bünning (1936) and later elaborated by C. Pittendrigh and others. Working with a *Drosophila* species, Pittendrigh found that this organism could respond differentially to LD vs. SD as a function of whether luminance does or does not make an incursion into what he termed the photoinducible phase of the animal’s intrinsic rhythmicity (It makes such an incursion in SD, it does not in LD [Pittendrigh, 1972]). External coincidence models have been explored at length and remain viable (Saunders, 2024). We may retain the term photoinducible phase for that part of the animal’s intrinsic rhythm on which light—*or rather luminance with the specific physical characteristics of a crepuscule*—lands or not. However these seasonal pioneers, so far as know, did not consider the variable of *duration* as regards the overlap (when it occurs) of the late crepuscule with the end of sleep. Nor did they scrutinize for seasonal relevance the *internal organization* either of sleep or of a crepuscule. What exactly qualifies as a crepuscule? And what exactly is the organism up to in late sleep that might render that particular stage critical for recognizing and engaging with SD?

We should begin by being clear about the baseline against which the animal in the wild measures photoperiodic change. Traditional laboratory protocols addressing this problem involve switching seasonal animals from a fixed LD to a fixed SD environment. Such studies have clarified that individual species have a particular scotophase duration any day exceeding which they count as a SD (for hamsters, this is about 11.5 h). But this fosters the notion that animals in their naturalistic setting work with a similar backstop-like value lasting hours. While such a backstop capacity may have utility in certain cases, animals in the wild routinely summon a more subtle instrument. For they effortlessly measure the degree to which the current scotophase differs from that obtaining 1 day earlier. In temperate zones, this difference changes through SDs by an amount varying from seconds just after the summer solstice up to about 4–7 min (depending on latitude) when changing most rapidly at the autumn equinox, then descending on the down side of the same curve until the winter solstice. How, in the graded day-over-day case, could organisms measure a change measurable *in minutes* as opposed to the hours of the backstop value? That laboratory animals *partially* approximating the graded case by being passaged through intermediate-duration photoperiods integrate such change more effectively than those treated with a fixed LD vs. fixed SD regimen (Prendergast et al., 2000) corroborates the notion that they have evolved to deal primarily with sidereal change as it unfurls with a day-over-day difference measurable in minutes. This means that we ought carefully to inspect the graded, i.e., the day-over-day case, as concerns both the sidereal side (i.e., the crepuscule) and the organismal side (i.e., the animal ‘reading’ it).

On the organismal side, mammals regularly assemble in homeostatic fashion a single maximally developed REM episode at the tail end of sleep (Benington and Heller, 1994; Sawada et al., 2024). In this context, homeostasis means that REM sleep (or rather NREM in association with REM sleep) builds on itself as the rest phase progresses, perhaps through the release of some mediator that lowers the threshold for the next episode. Thus the final REM episode tends to involve the greatest degree of *REM density* (e.g., Takahashi and Atsumi, 1997). Rapid-eye-movement density is a measure of the extent to which a REM sleep episode (which has tonic and phasic components) is populated by the purely phasic aspect which, of course, includes the REMs after which the entire episode was named. Students of the EEG measure REM density in one of two ways: they give either the average duration of a phasic REM episode (each burst of which may last up to a few minutes at most) or the fraction of time that a given tonic REM sleep episode such as the concluding one (which in the case of animals that consolidate sleep may last 45–90 min) is occupied by the phasic genre. We consider the duration of phasic REM bursts *in the final REM sleep episode*, i.e., its REM density, to have a photoperiodic rationale. We have two reasons for thinking so. First, the duration of a given REM burst may qualify as an interval timer (i.e., as distinguished from a circadian timer), a formal property long ago invoked as necessary to photoperiodism even in the absence of testimony to its physical makeup (e.g., Silver and Bittman, 1984). Second, researchers in mood disorders over 40 years ago began to document increased REM density as highly characteristic in two senses of the BD patient: he or she displays greater REM density compared to a control group and generally greater REM density when symptomatic than when not (e.g., Gold and Sylvia, 2016; Zangani et al., 2020). Incidentally, none of these researchers, including those writing after the formal recognition of SAD (Rosenthal et al., 1984) commented on any possible relationship between the REM density measure they found characteristic of the BD patient and photoperiodism at large. On this question, we part ways decisively with those researchers: we construe the REM density issue as centering in photoperiodism as such, a normative physiological process, not in its human remnant; the trend toward increased REM density may survive in that remnant and may prove useful as a source of evidence but that is not where it originated.

Here as elsewhere, anatomy helps. In the Syrian hamster and rat, the ipRGCs projecting through the LRHT arise almost exclusively in the superior temporal quadrants of the retina, indicating that these ipRGCs gather photic stimuli arriving from the inferior nasal visual fields (Johnson et al., 1988; Leak and Moore, 1997; Canteras et al., 2011). Now in REM sleep (at least in cats [Márquez-Ruiz and Escudero, 2008] and rats [Sánchez-López and Escudero, 2011]), the visual axes of both eyes, controlled proximately by motor neurons in the abducens and oculomotor nuclei, display a net tonic near-convergence and depression, implying attention to *something* in the inferior nasal visual fields; these tonic preferences are interrupted *but not fully corrected* by the supervening phasic REMs, e.g., in the vertical plane the eyes making REMs “very rarely” pass the center of the orbit (Márquez-Ruiz and Escudero, 2008, p. 3468). In other words, in REM sleep the *predominant* position of the globes is such as to direct photic information to the very ipRGCs innervating two at least of our candidate seasonal loci (the DMN and the LHA). Thus REM sleep may be driving the eyes (and through them select brain circuits) to attend in some fashion *not to a specific object but to a quality diffusely affiliated with a field, more precisely with the inferior nasal visual fields*. That quality is very likely luminance. Incidentally the fact that the axes do not *perfectly* converge in the REMs of sleep—they miss by some 15–30 degrees—has been entered as evidence against their foveating a dreamt object (Zhou and King, 1997). Although it might have been more precise to state that this failure of *perfect* convergence counts against strictly defined foveation of *any* object, not just a dreamt one, *imperfect* convergence is rather well-suited to intra-REM sleep sensing of background luminance. Interestingly, an orientation toward the ground in a phasic REM episode (as is *very roughly* the case for an animal in REM sleep) is compatible with the observation (Berry et al., 2023) that luminance is collected and averaged more accurately from a sweep of the proximate environmental floor than from elsewhere. While Berry et al. instance such “sweeping” done consciously in the animal’s waking phase (i.e., by scanning saccades while foraging), much else in their paper seems more relevant to the extraocular movements of sleep (i.e., REMs). For example, they show, as had others (Canteras et al., 2011), that the ipRGCs population differentially expressed in the dorsal retina projects among its other targets to the supraoptic nucleus (SON) including its so-called perinuclear zone (hereafter, peri-SON). Yet insofar as that peri-SON houses large cholinergic neurons representing an easily overlooked component of the magnocellular nuclei of the basal forebrain (Wang et al., 2015), it is surely activated to a greater degree in REM sleep vs. waking (Vazquez and Baghdoyan, 2001). Our tentative conclusion is that animals invented REMs in part to capture some aspect of luminance.

But which aspect? To answer that question, we turn now to the internal organization of a crepuscule. This clearly involves smooth ramping of illuminance through an extraordinary crescendo/decrescendo of values (as many as 11 orders of magnitude: Pugh et al., 1999). (If the seasonal animal is diurnal, then it sleeps in the dark and toward the end of sleep in SD it perceives a *crescendo* of luminance values from barely perceptible to maximal. If it is nocturnal, then it sleeps in the light and toward the end of sleep deals with a *decrescendo* of crepuscular luminance. The wiring diagrams for these two possibilities need not differ much). Such a degree of organization raises the question whether a given retinal and/or brain circuit specializes in processing such smoothly varying or so-called *luxotonic* signals. Interestingly, an early example of likely sensitivity to a luxotonic signal is available in the tunicate *Ciona*: a major trigger for metamorphosis in the *Ciona* tadpole involves exposure to twilight, a signal processed by coronet cells in its proto-hypothalamus (Lemaire et al., 2021). These cells are so named because they share morphological features with coronet cells in the saccus vasculosus of nontropical fishes, in which animals this organ has an established photoperiodic role (Nakane et al., 2013). That coronet cells in both *Ciona* and fishes express melanopsin (Lemaire et al., 2021) suggests that they represent early essays in circuitry involving ipRGCs. (Another chordate, *Amphioxus*, displays photosensitive cells that also provide a link via melanopsin from invertebrate to vertebrate ipRGCs [Koyanagi et al., 2005]). Now ipRGCs in mammals display an affinity not for random transitions amongst the many orders of irradiance to which they are sensitive but rather for those that take place naturalistically within crepuscules, i.e., luxotonically (Storchi et al., 2015). True, such sensitivity also occurs in newer and “higher” brain strata: thus Sabbah et al. have discovered it within the PFC—but as a result of that region’s engagement, through the so-called perihabenular region, with ipRGC fibers (Sabbah et al., 2022; Weil et al., 2022; Zangen et al., 2024). (A case is made later for the inclusion of the habenular region, one that surely precedes mammals [Boutet, 2017], in our seasonal module). Thus we may not need to look further than the ipRGCs traveling through the LRHT to find a system that specializes in sensing luxotonic signals when they come to the attention of the animal. Indeed we propose that ipRGCs with that capacity along with late-sleep REMs have evolved *specifically* for the task of recognizing and harvesting *that kind* of luminance signal and not others. (For simplicity, we omit for now any discussion of a second dimension along which crepuscular signaling varies smoothly, namely the wavelength of light gathered by the observing animal as twilight progresses). For an unvarying pattern of luminance, the kind, for example, available to the animal during REM sleep episodes *other than* those in the two crepuscular intervals at each end of sleep, tells it nothing about whether photoperiod is changing—and doing so luxotonically.

Why, given that both the first and second crepuscules possess luxotonic organization (regarding which they display mirror symmetry), have we claimed that the seasonal organism scrutinizes for photoperiodic evidence the *second* crepuscule only? Such a claim follows in large part from what we believe to be the foundational importance for photoperiodism of sleep homeostasis—that mechanism, introduced earlier in this section, by means of which the sleeping animal distributes its REM episodes asymmetrically, i.e., building gradually toward maximal REM density in the concluding REM sleep episode. Strictly speaking, photoperiodism, by selecting some basis other than that afforded by sleep homeostasis, could have evolved otherwise and may have done so in a very limited niche. At least one extant reptile regularly places two reptilian equivalents of REM sleep (termed the S2 state) symmetrically, i.e., once at the beginning and once at the end of sleep (Libourel et al., 2018). This is the Argentine giant tegu *Salvator merianae*, native to much of South America and clearly seasonal (Zena et al., 2019). Yet entering REM sleep *early in the rest phase* exposes an animal to increased risk from predators (Lima et al., 2005; Lesku et al., 2008). The predation risk may not have posed a major problem for the giant tegu, the male of which may grow to over 4 feet in length and both genders of which are more reliably predator than prey. However this exception proves the rule: it is almost surely the predation risk that has caused evolution to discourage, in almost all other species, the placement of S2 or REM sleep early in sleep. Putting the matter positively, evolution has decisively favored a form of sleep homeostasis that loads heavily on increasing REM density at the end of sleep. While this does not *necessarily* mean that sleep homeostasis evolved under photoperiodic selection pressure, such likelihood follows from the seasonal utility of the final, asymmetrically developed, S2 or REM sleep episode.

## 6 Do luxotonic signals speak preferentially to astrocytes?

As noted above, smaller temperate-zone organisms first give neuroendocrine notice of having initiated the SD response with an elevation of vasopressin and oxytocin by roughly the middle of August (see section 2). During that month scotophase is growing from 1 day to the next by an approximate mean value of 2 min (at the latitude of Portland, OR—but this day-over-day change is not latitude-dependent in the strict fashion obtaining with the absolute duration of scotophase). Thus 2 min may represent the approximate lowest *effective* day-over-day change capable of triggering the organismal SD involutional response; its obtaining in August fits with the notion that the animal in its naturalistic setting *initiates* its involutional response well ahead of winter, not waiting until that change comes closer to its maximum 4–7 min at the autumn equinox. But then why should a roughly 2-min interval in particular serve to trigger the very beginning of the animal’s SD response?

In casting about for a possible equivalent on the organismal side to the two-minute sidereal threshold, we have been drawn to the astrocyte. This cell type displays a well-known specialization in AG the seasonal modulation of which features prominently in photoperiodism. Indeed as proposed elsewhere, activation of the AG pathway distinguishes the SD animal, cutting across many different varieties of photoperiodic organization (e.g., nocturnal versus diurnal animals, obligate versus facultative hibernators, and infrahuman species versus human beings) (Raitiere, 2020). Now the astrocyte is notorious for computing data much more slowly than neurons which work generally in the millisecond range (e.g., Vardjan et al., 2016). Thus astrocytes require a minimum of approximately 2 min after NE or epinephrine (E) stimulation (a) to import a quantum of glucose from the intravascular or the extracellular space (ECS) and polymerize it with the existing intracellular storage form of glucose, i.e., glycogen polymers, already stationed in granular form inside this cell type, *while at precisely the same time* liberating the same *amount* of glucose (although different molecules) from the other ends of the same polymers; (b) to rush the glucose thus loosened (which, remember, has been intracellular all along) through the 10 sequential enzymatic steps of AG that yield lactate as its end-product; and (c) to export the resulting lactate into the ECS for dispersal through the astrocytic syncytium (Prebil et al., 2011; Horvat and Vardjan, 2019; Fink et al., 2021). R. Zorec’s Ljubljana group, scrupulous students of astrocyte physiology in contexts quite independent of seasonal matters, have ascertained the time constant for the process just outlined to be 116.2 s after NE stimulation and 115.9 s after E stimulation (Prebil et al., 2011). Not exactly two organismal minutes but pretty close.

We cannot proceed further in our review of the AG pathway without alluding to the well-known controversy surrounding its fate once exported into the ECS. Pierre Magistretti and colleagues have long argued that lactate in the ECS provides fuel to neurons; this is the ‘astrocyte-neuron lactate shuttle’ (ANLS) (Magistretti and Pellerin, 1999). An alternative position centers on the discovery that lactate once transported into the ECS has a signaling function by virtue of interacting with a specific receptor embedded within the plasma membrane of the astrocytes that produced it (Tang et al., 2014; Vardjan et al., 2018). Now any mechanism whereby a molecule *previously* released into the ECS signals through a receptor on the cell that produced it in such fashion as to enhance *the next round* of its own production cycle (as does lactate in the LC-based NE system: [Tang et al., 2014]) possesses memorial value on its face. Although not yet demonstrated for the medullary NE system, this represents exactly the species of tight feedback loop that must obtain in order to integrate a memory not of an isolated scotophase but of a series of these.

Reformulating the conditional nature of the SD response, we may say that the secure anchoring of SD perception takes place when two vectors, one organismal and the other closely tracking a sidereal value, merge. The organismal vector includes the pontogeniculooccipital (PGO) waves emanating from the so-called REM sleep executive area in the sublaterodorsal nucleus (SLD, termed nucleus pontis oralis in some species), a locus tightly linked with the PPTg/LDTg cholinergic neurons (Datta et al., 1998). As we have seen, the vector deriving from the sidereal environment drives luminance-related activity within the LRHT pathway. Now these two vectors may initially be viewed as functionally orthogonal to each other. Yet their footprints overlap contingently. Thus the PGO wave footprint, which includes amongst its *possible* targets the LHA and much of the medial hypothalamus (Datta et al., 1998), reaches maximal overlap with the ipRGC-afferented territory in SD.

But note now a subtle change in our understanding of the SD dynamic. As formulated up to this point, perception of the luxotonic quality of crepuscular luminance might be construed as a passive process on the part of the animal. But if the inspection of the sidereal environment by the REM sleep apparatus takes place within each culminating REM sleep episode, *then that apparatus must be working as an active probe of the luminance environment—and doing so all year long*. One consequence of this activist view of REM sleep involves the plasticity of sleep in SD. Well documented as one sign of winter depression in human beings with SAD/BD (Rosenthal et al., 1984), hypersomnia in the psychiatric literature often figures as a symptom, one that has caused the depressive phase of SAD/BD to be classified within a nosological category that originated independently of seasonal interests, namely “atypical depression” (Davidson and Thase, 2007). Yet this largely pathological view of the extension of sleep as applied to the SD organism misses, we think, its core physiology.

In large part a function of melanin-concentrating hormone (MCH) neuronal projections from the LHA to the infra-hypothalamic brainstem (Jego et al., 2013; Vetrivelan et al., 2016; Pascovich et al., 2021), the extension of REM sleep surely develops *subsequent* to the recognition of SD. Longer phasic REM sleep implies an increasing density of two-plus minutes of phasic REM episodes (as opposed to an increasing frequency of such episodes unsorted for duration), That density, we think, grows in synchrony with the progression of SD. Upon transmission from the LHA to the brainstem, a string of two-plus minute REM bursts serves as a signal to elongate REM sleep. This differs somewhat from the view of Sapin et al. (2010), who view instructions from the LHA to the brain stem as in effect “maintain REM sleep.” What is new here is not the suggestion that orexin vs. MCH LHA neurons drive REM sleep down vs. up respectively—the Lyon group and others saw to that (Verret et al., 2003; Hassani et al., 2009; Luppi et al., 2013)—but rather the proposal that the ratio of orexin vs. MCH neural activity in the LHA varies as a function of photoperiod. But how does this region interact with the remainder of the seasonal module, especially as regards its vocation in SD?

## 7 The seasonal module in autumn and winter

### 7.1 The engagement of the dopaminergic A10 and A10dc dopamine neurons in (and by) the shorter days of the year

**Figure 3:**
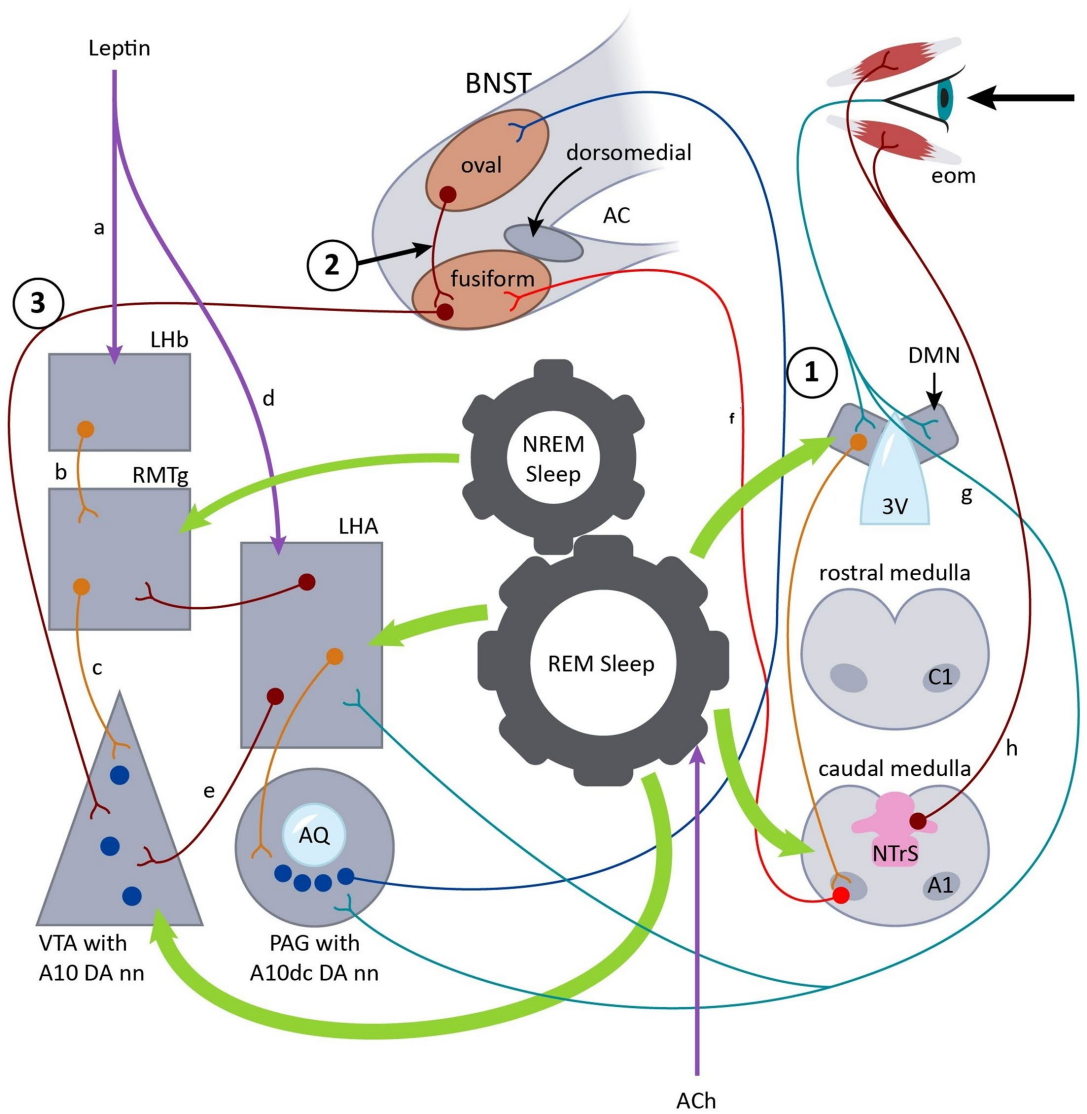
The seasonal/REM sleep module at work. Here we combine two approaches, one *anatomic* and the other *functional*, in order to clarify the role of REM sleep in photoperiodism. The *anatomic* approach centers primarily on the trineuronal series illustrated by the three neurons labeled 1, 2, and 3 (numbers placed in circles here and in Figure 4). As discussed here and in section VIIIA of our text, this trineuronal series comprises a complex trans-BNST pathway through which the A10dc DA neurons of the vlPAG (modified by luminance) modulate the larger A10 DA group in the VTA. Thus DAergic neuron 1 includes two subsets: an inner ring immediately adjacent to the Aq and somewhat larger neurons scattered through the DRN. The former subset is surely modified by melatonin in the CSF; the latter, by luminance data supplied by retinal afferent g. Both of these subsets collectively contribute to the long axon of neuron 1 which terminates in a concentrated fashion upon dopaminoceptive neurons in the oval subnucleus of the BNST. Neuron 2, enclosed within the BNST, serves as a GABAergic bridge between the oval subnucleus, where it originates, and the fusiform subnucleus, in which it terminates. Neuron 3 is a glutamatergic output neuron located in the fusiform subnucleus; taken collectively, it terminates massively in the VTA. We do not address the question whether it makes synaptic contact directly with the DAergic A10 neurons here or rather indirectly by way of intra-VTA GABAergic neurons linking up with them (or utilizes each in different seasons). One advantage of the trineuronal trans-BNST pathway is that it is open to modulation at least two of its nodes (the PAG and the BNST) by molecules transported by volume transmission. (This will be discussed in conjunction with Figure 4). Full citations involving the trans-BNST pathways 1, 2, and 3 as well as to the habenular connection to that trineuronal chain are given in section 7.1 of our text. As for pathway f from the A1 noradrenergic neurons to the fusiform BNST subnucleus, see Shin et al. (2008). The second, *functional*, approach involves the contribution of non-REM and REM sleep as indicated by the two geared structures placed at the center of the figure. The broad-brush arrows emanating from each of these two structures are meant to suggest the multiple means by which these sleep phases work in and through many components of the module. The arrows depicted touch upon only four of the most important loci within the module, namely the LHA, the VTA, the caudal medulla (including the NTrS), and the DMN. While it would be impossible to indicate all of the pathways by which REM sleep achieves its seasonally relevant effects, we list here a number of studies that discuss its role within the four indicated regions. *For the LHA*, see Clément et al. (2012) and Feng et al. (2020) (a helpful if somewhat contrarian view of the contribution to REM sleep of orexin neurons in the LHA). *For the VTA*, see Dahan et al. (2007) (discussed in section 7.2). *For the DMN*, the links to REM sleep seem at first elusive. One may begin by noting that all preoptic area neurons, many of which have been implicated in sleep (Arrigoni and Fuller, 2022), project in particular to its anterior portion (Thompson and Swanson, 1998). Further, the SD animal is differentially sensitive to leptin (Rousseau et al., 2003); leptin receptor neurons are richly expressed in the DMN (Laque et al., 2013); and microinfusion of leptin, e.g., into the VLPO (Ramírez-Plascencia et al., 2022), generally augments NREM and REM sleep. *For the caudal medulla including the NTrS*, see Gutierrez Herrera et al. (2019), Schott et al. (2023) and Mir and Jha (2024). Abbreviations not previously noted: ACh, acetylcholine; AP, area postrema; Aq, cerebral aqueduct; DRN, dorsal raphe nucleus; eom, extraocular muscles driving REMs; LHA, lateral hypothalamic area; LHb, lateral habenula; NPap, nucleus papilio (enclosed within NTrS); NTrS, nucleus of tractus solitarius; PVN, paraventricular nucleus of hypothalamus: RMTg, rostromedial tegmental nucleus; VLPO, ventrolateral preoptic area; VTA, ventral tegmental area.

It is time to propose a hypothesis whereby the SDs of autumn and winter may incrementally drive our seasonal module into hypoarousal (whether torpor or hibernation). By way of doing so, we add two interrelated loci, namely the lateral habenula (LHb) and, downstream from it, the rostromedial tegmental nucleus (RMTg) to our seasonal module as originally envisaged. With these additions we arrive at Figure 3. As will be seen, the LHb and the RMTg are well positioned to *downregulate* midbrain DA systems on a seasonal scale. REM sleep remains in the background as a sampling device for changing luminance. (We omit discussion of another such agent of change, leptin, sensitivity to which increases markedly in SD [Rousseau et al., 2003]: its neuroprotective/neurogenetic capacity and therefore its aptitude for bringing about seasonal modification has been well documented [e.g., Regensburger et al., 2023] and need not be elaborated here).

We focus now on seasonal plasticity within the two midbrain DA loci of greatest seasonal interest: the VTA (containing the DA A10 neurons) and, within the vlPAG, the cluster of DA neurons known as the A10dc group (where ‘dc’ stands for *d*orso*c*audal extension of the more widespread DA A10 group). As we have proposed elsewhere (Raitiere, 2022), luminance-sensitive ipRGCs directly and indirectly modulate the DA A10dc group. (The case for direct impingement remains an extrapolation from multiple studies demonstrating retinal afferentation of serotonergic cells in the dorsal raphe nucleus [DRN] within which many of those DA A10dc cells are scattered [Shen and Semba, 1994; Fite et al., 1999; Fite and Janusonis, 2001; Hattar et al., 2006; Luan et al., 2011; Ren et al., 2013]). Here we expand that proposal by suggesting that the A10dc neurons in turn bring influence to bear on the larger A10 group rostral to it in the VTA. Although a unidirectional, dense, and partly DAergic connection between the vlPAG and the VTA is available (Omelchenko and Sesack, 2010), we bring forward, for its seasonal utility, an indirect but eventually robust link passing through the BNST portion of the CEA before projecting compellingly upon the VTA (see serial pathways 1, 2, and 3 in Figure 3). (For neuron 1, see Hasue and Shammah-Lagnado, 2002, and Meloni et al., 2006. For neuron 2, entirely intra-BNST, see Dong et al., 2001. For neuron 3, see Jalabert et al., 2009). Also mediating between luminance and the DA A10dc cells is melatonin: by virtue of immediately adjoining the cerebral aqueduct where levels of melatonin markedly exceed those in plasma (Reiter et al., 2014; Tan et al., 2016), one subset of the A10dc cells, expressing vasoactive intestinal polypeptide (VIP) along with DA (Dougalis et al., 2012), is likely to be differentially sensitive to the LD vs. SD difference in the duration of the melatonin pulse. Given that VIP release is a strong feature of REM sleep (Kohlmeier and Reiner, 1999; Ahnaou et al., 2006; Hu et al., 2011), that particular subset, figuring in the VIP projection to the BNST (Petit et al., 1995), very likely comes on line specifically inside REM sleep. In sum, the DA A10dc → BNST → DA A10 linkage is proposed as a critical substrate for the SD response. Finally, the lateral habenula, an ancient structure with its own well-documented links to luminance (Boutet, 2017), has multiple means of influencing our trans-BNST trineuronal chain (see below). It should not be surprising that ablation of the middle locus in this chain (i.e., the anterior BNST) markedly impairs the SD response (Raitiere et al., 1997).

However, any claim for the A10dc cells as subserving involution exclusively comes up against the opposite position articulated by the Saper group in a frequently cited 2006 study. Here Lu et al. (2006) claimed the DA vlPAG cells as a key DAergic substrate for waking arousal. To be sure, the authors did not link their species of A10dc cell-driven arousal to photoperiodism—yet their paper offers an excellent map for the brief but potent hyperarousal phase of SD (see section 3.1). *But to leave the matter there ignores the potential for polarity reversal within the same network.* Indeed 3 years after that publication (and thanks in no small part to two of its coauthors, T. C. Jhou and Clifford Saper), we began to learn much about another CNS locus that specializes as something of a brake on the VTA, namely the RMTg (Jhou et al., 2009a, 2009b; Kaufling et al., 2009; Lavezzi and Zahm, 2011; Barrot et al., 2012; Rossi et al., 2021; Jhou, 2021; Cui et al., 2025). Given its capacity for *downregulating* the DA neurons of the VTA (Balcita-Pedicino et al., 2011; Barrot et al., 2012; Jhou, 2021; Cui et al., 2025), the RMTg, linking through pathways a-e of Figure 3 with our trineuronal trans-BNST circuit (Jhou et al., 2009a, 2009b; Kaufling et al., 2009), may contribute significantly to the involutional SD phenotype.

Now the metaphor of the brake as applied to the GABA neuron-enriched RMTg (Barrot et al., 2012) may be misleading insofar as it evokes only the temporal domain of ‘fast’ GABAergic action. The question here, incidentally, is not so much *whether* the trineuronal and trans-BNST pathway serves as a conduit for some agent or agents of the downshifting to involution; it seems clear that it somehow does when nature, via its SDs, is doing the experiment. The issue is *how* nature, working stealthily, one REM sleep interval at a time, achieves the behavioral opposite of what J. Lu and colleagues accomplish by optogenetically exciting the DA system portrayed collectively by our neuron 1 (Lu et al., 2006).

**Figure 4:**
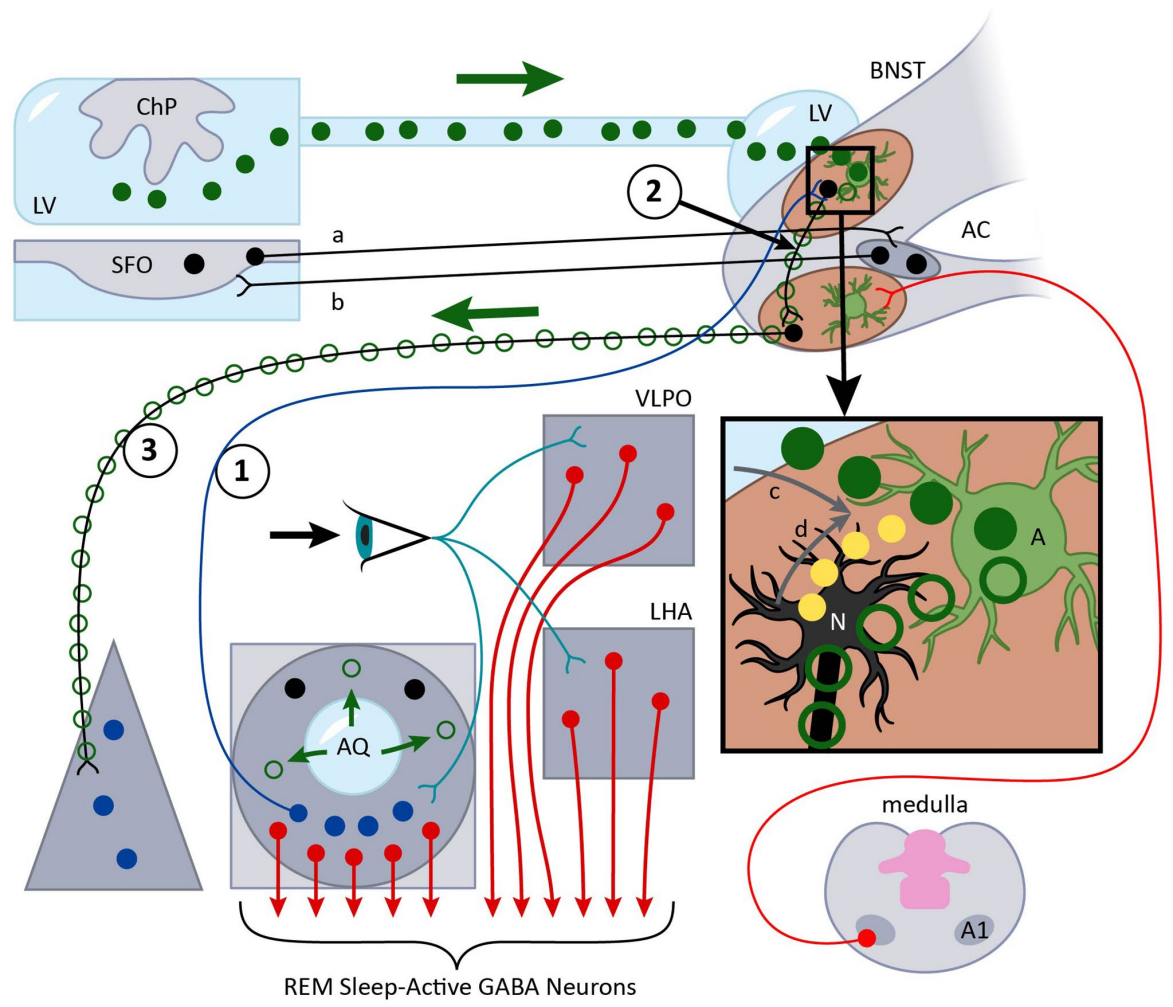
Contributions of lipid transport and signaling within seasonal module. Here we illustrate what we believe to comprise the relevance of lipid transport to seasonal physiology and specifically to the BNST component of the seasonal module. We have already discussed the trineuronal series consisting of neurons 1, 2, and 3 (illustrated here and in Figure 3) and will mention it here only insofar as it bears on lipid metabolism and transport. An additional example of hard-wired transmission, though one with ties to volume transmission, involves the reciprocating link, the components of which are labeled as a and b here, between the dorsomedial subnucleus of the BNST and the subfornical organ (SFO; see Swanson and Lind, 1986 and Dong and Swanson, 2006). We begin with the volume transmission component of lipid transport. This form of communication includes trafficking through cerebrospinal fluid (CSF) and interstitial fluid (ISF) of a host of molecular mediators, many included within membrane-bound extracellular vesicles (ECVs). Lipids feature in ECVs both as enclosing membranes and as a portion of their contents. In the figure, ECVs are represented by green circular bodies emanating primarily from the choroid plexus (ChP). From that point CSF carries these unidirectionally through the ventricles. Transporting a number of molecules implicated in neuroprotection and neurogenesis (Trueta and De-Miguel, 2012), the ECVs are able to move from CSF into brain tissue at multiple ventricle/neuropil interfaces. These include, at the lateral ventricle/basal forebrain interface, the BNSTov; at the third ventricle/hypothalamus interface, the DMN; at the cerebral aqueduct/vlPAG interface, the DA A10dc group immediately adjacent to the aqueduct (discussed in section 8.1); and at the fourth ventricle/caudal medulla interface, the dorsal vagal complex (not labeled as such in this figure). Thus at the first, second, and third of these interfaces, our diagram depicts ECVs as passing from CSF into brain parenchyma. Apart from accepting mediators originating within CSF, the BNST houses its own form of lipid trafficking involving movement between astrocytes and neurons. This species of trafficking has been well studied by the Bellen group in particular (Liu et al., 2017); although that group has not anchored its version of the ANLS within a given anatomic circuit, we take that step in sections 7.1-7.2, aligning it within the BNST. Thus, with modifications, it becomes the focus, here in Figure 4, of the expanded box. As per the Bellen group, neurons place the lipid waste products of their own activity into vesicles which they then convert for export as ECVs (pathway c here). Once in the extracellular medium, these ECVs, converging with those deriving from CSF (pathway d), are then imported into astrocytes. Here we diverge from Bellen et al.: astrocytes, we propose, detoxify the contents of material imported through pathways c and d, add their own molecular mediators—primarily neurotrophic factors—and return the ECVs, now cleansed, to the extracellular medium. Albeit briefly, the seasonal roles of neurotrophic factors will be addressed in the text.

We may approach this question by recalling that the ultimate cause of seasonal involution is generally held to involve survival—that of the individual as well as of the species (see section 1). Both kinds of survival in turn rely on neuroprotection and neurogenesis. Significantly, hypothalamic neurogenesis in seasonal animals peaks in SDs whether they are SD or LD breeders (Batailler et al., 2018). We have discussed the role of lactate, which itself has significant neuroprotective potential (Wu et al., 2023), in potentiating the SD response. By exploring one important lactate-related mechanism, mentioned only in passing thus far, Magistretti’s ANLS, we may get some sense for one means by which lactate could work *slowly* in the CNS. In brief, this concerns its promotion of molecular transport. Magistretti made one such form of transport, that of lactate into and out of neurons and astrocytes via monocarboxylate transporters, an intrinsic part of his ANLS (Pellerin and Magistretti, 2012). But there is another genre of molecular transport: that of proteins, lipids, and smaller nucleic acids (e.g., microRNAs) along axonal processes such as those linking forebrain to brainstem. Since axonal transport is now understood to be powered by AG (Mc Cluskey et al., 2024), these two genres of transport may conceivably be combined into a single apparatus. We present our speculation about such an updated ANLS-*like* shuttle in Figure 4. Note that this device finds support in recent work on lipid metabolism, now understood to have a decisive influence on the seasonal calendar (e.g., Levine et al., 2025). By way of demonstrating the ancient connection between neuroprotection, lipids, and transport, we mention recent work on brain fatty acid transport protein (FATP7) which regulates photoreceptor neuron survival in *Drosophila* (Dourlen et al., 2012) and correlates closely with *increased* sleep from *Drosophila* to human beings (Gerstner et al., 2017, 2023; Needham et al., 2022; Flores et al., 2025). We come closer to understanding how complex forms of transport through our trineuronal chain may be involved in securing involution and thus survival. And we shall offer a conjecture as to what precisely, carried through that particular chain over weeks of SD, may bring the organism to that end.

### 7.2 Why do the A10 neurons burst-fire in REM sleep?

Let us turn for a moment to an ancestor of the mammalian hypothalamus, the pars intercerebralis of insects (Hasebe and Shiga, 2021a, 2021b). The larger neurons of the pars intercerebralis of the green bugs *Riptortus pedestris* and *Plautia stali* fire at high frequency under SD conditions. Such an elevated firing frequency is required here as well as in the mammalian line for the release of peptides which in the case of the green bug drive the photoperiodic phenomenon of diapause (Hasebe and Shiga, 2021a, 2021b). Might firing frequency sufficiently elevated to cause neuropeptide release play a role in mammalian photoperiodism including REM sleep? A firing pattern meeting criteria for bursting (one end-point of high-frequency firing: Shao et al., 2021) transpires with regard to a provocative REM sleep-related phenomenon that remains as of yet poorly assimilated to REM sleep orthodoxy. This is the bursting *in REM sleep* of VTA DAergic neurons (i.e., the A10 group discussed in the previous section) (Maloney et al., 2002; Dahan et al., 2007). Why should VTA DA neurons in the midst of a REM sleep episode switch to burst mode—a firing pattern, to be sure, which the animal *when fully awake* reliably displays upon perceiving a salient and likely rewarding item (Schultz, 2002)? To explain VTA bursting in REM sleep, some investigators have borrowed a rationale from the waking case: insofar as DA can drive synaptic plasticity, it could in REM sleep be consolidating memory for one of those fetching waking-phase items via the VTA-hippocampus reciprocating loop (on which see Lisman and Grace, 2005). This, the strategy adopted by Dahan et al. (2007) themselves in order to explain their discovery of VTA neuronal bursting in REM sleep, seems at first glance unexceptionable. However as they acknowledge, VTA DA neuronal bursting in REM sleep displays some differences vs. the active-phase response to reward: whereas most studies of VTA DA burst firing in response to reward document one time-locked burst (reviewed in Schultz, 2002), in sleep these DA neurons switch to bursting *for the entire duration of a REM sleep episode* beginning 10–20 s before its onset. *We submit that the bursting pattern in sleep suggests a high degree of faithfulness to REM sleep itself* (to utilize for the moment an admittedly vague expression) rather than to a specific item being replayed within it. Can we account for such DA neuronal bursting almost exactly coterminous with a phasic REM sleep episode in a manner that is *not* related to memory for reward or, for that matter, to anything else connected to waking life?

It is well established that a bursting pattern in DA neurons results in massive release of the monoamine especially when paired with blockade of its reuptake (Floresco et al., 2003). Precisely such blockade of the major DA transporter *in the DMN*, the high-capacity, low-affinity organic cation transporter (OCT, especially the OCT3), is achieved by infusion of exogenous GCs into the same hypothalamic region or by stress-induced elevation of endogenous GCs in the same region (Gasser et al., 2006; Gasser et al., 2009; Gasser and Lowry, 2018; Gasser, 2019; Holleran et al., 2020; Benton et al., 2021). Now Lowry et al. (2003) took stress to be the likeliest context for their finding—especially since restraint stress had been found frequently to increase GCs which in turn blocked the reuptake of monoamines (although not always in both genders or in all rat strains). Intriguingly, Lowry had been drawn to the DMN in particular because of an early interest in photoperiodism (e.g., Lowry et al., 1996, 1997); indeed he noted that a region within or bordering the DMN could be construed as having developed from the paraventricular organ (not to be confused with the PVN of mammals), a major player in reptile and bird photoperiodism (Lowry et al., 1996, 2003). He also knew from this early work that certain monoamines are not infrequently high *at baseline*, i.e., exclusive of stress, in the DMN in those photoperiodic animals (Lowry et al., 1996, 1997). In the seasonal case as in that of stress, GCs may well have been blocking the OCT and thereby augmenting monoamines in the DMN—but according to a logic differing from that of stress.

As we noted in passing earlier (see section 4 and Figure 2), GCs are increased and generally phase-advanced in the SD response. It is submitted that such augmentation of the HPA axis in SD has little or nothing to do with stress. Thus In the *unstressed* Syrian hamster, 18 days of SD treatment results in elevation of the GC pulse along with increases in the widths of the adrenal zona reticularis and glomerulosa (Lance et al., 1998). In the *unstressed* Fischer 344 rat, 14 days in SD witnesses the emergence of an increased and phase-advanced GC pulse as well as increased sensitivity of the adrenal cortex to ACTH in contrast to LD controls (Otsuka et al., 2012). Incidentally, the BD patient’s GC phase-advance and increase recapitulates that of the animal in SD up to the autumn equinox and is not contradicted at all by the well-documented later collapse of the HPA axis in SAD patients *in winter proper* (Raitiere, 2022). The reason for the autumnal SD increase in the animal’s HPA axis is unclear and the entire story cannot be explored here. Here is the short story: we have not one but two contexts for the increase of monoamines and GCs in the DMN. It is difficult to tease them apart especially since their anatomy overlaps. (Compare that of the stress response [Choi et al., 2007] to our seasonal module). Yet we may do so by consulting the location of the GC acrophase.

While augmenting GC output, stress does not modify the tendency of the GC pulse to peak just after awakening, a position justifying the classic description of its tasks in endocrinology textbooks in terms such as “getting ready, metabolically and otherwise, for the stressful day ahead.” Conversely, the early SD advance of the GC acrophase by some 2 h with reference to the more inertial sleep–wake cycle (Lemos et al., 2009) brings it roughly into the middle of the last and longest REM sleep episode of the day. (For a modest increase in GCs inside all REM sleep without reference to photoperiodism, see Machado et al., 2013; Machado and Suchecki, 2016). We suspect that only SD attracts the GC acrophase into sleep and, more precisely, into the last REM episode. There, it carries out, we believe, a number of tasks unrelated to stress. We mention three of these here: first, the GC peak in this location is deprived of the ability to waken the animal; i.e., the “cortisol awakening response” does not materialize in SD (Thorn et al., 2011). Second, the cortisol elevation inside REM sleep is accompanied by a distinct *anti-stress* effect, one likely mediated by prolactin (Machado and Suchecki, 2016). Third, that elevation inside REM sleep surely impairs the uptake capacity of the OCT3 just as surely as stress does, thus accounting for elevated monoamines in, of all places, sleep and for the entire duration of the REM episode. This gives substance to our admittedly vague notion, presented earlier, of fidelity to the REM episode as such rather than to an item within it. (Collapsing once again a long story, we suspect that those augmented monoamines, especially NE [e.g., Lazzeri et al., 2021] and DA [Di Nunzio M et al., 2025] underwrite production of neurotrophic factors such as brain-derived neurotrophic factor). Fourth, the shift of the GC peak into REM sleep likely accounts for its modification of the extraordinarily complex neurotransmitter portfolio within the CEA. Well documented as a consequence of exposure to cortisol within our seasonal module is activated neurotensin (NT) transcription; (see below). This brings us to that proposal about the particular agent that, transported from the vlPAG to the VTA in SD, is responsible for the seasonal animal’s pirouette into involution.

A number of anatomists including in particular Daniel Zahm have noted that a significant number of afferents converging on the VTA express NT (Zahm et al., 2001; Woodworth et al., 2018). Despite the extent and redundancy of this system, no solution to its core functionality has emerged. We now advance the hypothesis that its functionality turns ultimately on photoperiodism. More specifically, NT comprises at least one of the agents which, gradually disseminated in SD through the VTA from a surprising number of source loci, accomplishes our phenotypic reversal. Neurotensin has long been noted to possess both pro- and anti-DAergic effects with evidence indicating that the latter predominates in SD (Helwig et al., 2009). A significant subset of NT neurons also expresses the leptin receptor (Woodworth et al., 2018), the sensitivity of the ligand for which is markedly activated in SD (Rousseau et al., 2003). Given that NT neurons in the LHA and perifornical area are particular targets for direct GC actions (Cintra et al., 1991), we propose in the briefest compass the following means of admitting REM sleep to seasonal physiology: in SDs, attraction of GCs into the REM sleep interval empowers augmentation of NT within the neuronal meshwork converging on the VTA. Together with leptin, this represents a core component of the machinery that *incrementally* downregulates VTA DA function in SD. This is an eminently testable hypothesis. In any event, since our through-line is less the descent over SD into torpor and hibernation *per se* than the relationship of REM sleep to that process, we must conclude by focusing on the deeply organic nature of that relationship. We do so by summoning evidence from a previously unmentioned domain, that of the so-called deep brain photoreceptors. Long acknowledged in invertebrates, these molecules, directly sensitive to luminance and generally unrelated to retinal efferents, have now been documented in mammals in locations deep in the brain and yet superficial in the periphery (e.g., skin tissue [Ma et al., 2025]). One of these photoreceptors can speak to the manner in which REM sleep improves the SD animal’s discernment of photons.

## 8 Thermogenesis, QPLOT neurons, REM sleep, and violet light

Zhang et al. (2020) in the Cincinnati laboratory of Richard A. Lang demonstrated that mice can sense visual violet light (380 nm wavelength) through a deep brain photoreceptor, namely the opsin 5 variant of the opsin family, residing in the preoptic area (POA) of the hypothalamus. Furthermore they showed that violet light stimulation of the mouse POA opsin 5-bearing neurons strongly *suppresses* brown adipose tissue (BAT) thermogenesis. This finding cannot but recall one classic phenomenon involving the conjunction of sleep with suspension of BAT thermogenesis—namely, REM sleep. Curiously, Zhang and colleagues made no explicit mention of REM sleep. Yet they could not but be well aware—given their demonstrated expertise on the topic in other studies—that the POA, via two likely overlapping cell populations, has a well-known role in sleep. Thus in 2021 they identified the full genetic signature of a POA cell population, including the opsin 5 photoreceptor, which they termed QPLOT neurons, that both drives ‘quiescence’ and *suspends* thermoregulation (Upton et al., 2021). Here they noted that QPLOT neurons, partly by virtue of bearing leptin receptors (honored with ‘L’ in their acronym), likely had a role in seasonal physiology including torpor—but still not a word on REM sleep as such. They followed this up in 2022 with the discovery that a cluster of neurons immediately adjacent to the QPLOT group displays sensitivity to temperature but not to light—essentially the obverse of their QPLOT group (Díaz et al., 2022). Yet still they did not acknowledge the relevance of their discovery to REM sleep.

Why this group, in the papers in question, has been reluctant to name REM sleep as the pathognomonic example of ‘quiescence’ *plus actively inhibited* thermoregulation remains unclear. (Ignorance may be dismissed as an explanation). For what it is worth, we lodge the following guess: Lang and company must be well aware that the ultimate cause of REM sleep remains an enigma, one that has attracted its share of practitioners from the ‘soft’ sciences (e.g., practitioners of dream interpretation) and they may not have seen much value in meddling in it. That’s unfortunate—if only because their remarkable elucidation of QPLOT neurons fits rather well with the hypothesis presented here. Having suggested that the final REM sleep episode of the day represents that particular segment of the organism’s rest phase devoted to interrogating luminance, we now propose a means of reconciling subtraction of concomitant BAT thermoregulation with that process. To maximize the salience of luminance within this interval specifically, the animal must suspend sensitivity to any *other* genre of sensory input to the POA. High on the list of signals other than luminance presenting to the POA is temperature. In other words, having an opsin in its POA that turns off thermoregulation allows the animal to engage most accurately with the violet-rich end of the visible light spectrum—particularly in the crepuscules, the temporal unfolding of which by frequency includes a tranche of violet light. (Abundant outdoors, violet light is deficient indoors due to ultraviolet/violet light blocking materials in windows, eyeglasses, and indoor light sources [Kondo et al., 2025]). Its acuity aided by cancelation of the relatively noisy temperature signal, the seasonal animal in REM sleep can best calibrate its allegiance to the luxotonic moment. Having ‘read’ that cue, the animal will engage in torpor or hibernation no later or earlier than its internal calendar dictates.

## 9 Conclusions and future directions

We may now sum up our hypothesis by evoking a thought-experiment along the lines of Michael Corner’s: he imagined, for heuristic purposes, a prototypical animal with an active phase only. Corner and Schenck (2015) found remnants of such an organism early in both phylogeny and ontogeny. Evolution may have noted, as it were, that a few exceptional animals that somehow had stumbled upon a means of interposing a phase of *not moving* had a greater propensity for surviving winter than those continually moving; it may also have discerned that those surviving were distinguished not by absolute passivity but by the incorporation of a specific metabolic cascade they had perfected while stirring about. This was the generation of lactate through AG (which product we humans continue to produce lavishly when navigating consciously in the waking phase). Such an exceptional benefit of lactate-minus-motion could have initiated evolutionary pressure for developing a new epoch called rest—and, within that epoch, a more specialized interval yet, one rigorously emptied of almost all continuous movement apart from a few exceptions, primarily extraocular musculature. Here motor atonia entered what would become REM sleep and never left it.

We hope that future work will evaluate and test the view proposed here that an animal with a fully developed, REM sleep-inclusive, sleep–wake cycle fully independent of seasonal demands is an artifact. We see no need to invoke such an organism (or its useful but equally imaginary relative, the animal in the wild that experiences a run of fixed-photoperiod days). For we suspect that evolution tinkered early on *and simultaneously* with designing a rest-activity cycle for organisms *and* with preparing them for winter survival. Ascertaining when that dual process began is not the point and anyway may never be figured out. Rather, one task for the future will be to test the hypothesis that an animal’s sleep–wake cycle *as we have access to it nowadays* is already deeply imbued with seasonal imperatives. The many papers, for example, of the Lyon, Cambridge (USA), and other laboratories on the substantial contribution to REM sleep of MCH neurons in the LHA fall neatly into place with photoperiodism once we grasp the markedly elastic nature of the phasic REM sleep interval: the elasticity is there as a means by which to promote seasonal survival. Similarly, we may achieve greater insight into sleep homeostasis once we assess that process as linked inextricably to photoperiodism—as optimizing the animal both for the momentous final REM episode of the day as well as for the neuroprotective/neurogenetic potential stabilizing incrementally within it. We hope that our hypothesis may play some part in orienting interested students to such future tasks.

## Data Availability

The original contributions presented in the study are included in the article/supplementary material, further inquiries can be directed to the corresponding author.
